# Development of a Simple Setup to Measure Shielding Effectiveness at Microwave Frequencies

**DOI:** 10.3390/s24123741

**Published:** 2024-06-08

**Authors:** Emanuele Cardillo, Fabrizio Lorenzo Carcione, Luigi Ferro, Elpida Piperopoulos, Emanuela Mastronardo, Graziella Scandurra, Carmine Ciofi

**Affiliations:** Department of Engineering, University of Messina, 98166 Messina, Italy; ecardillo@unime.it (E.C.); fcarcione@unime.it (F.L.C.); epiperopoulos@unime.it (E.P.); emastronardo@unime.it (E.M.); gscandurra@unime.it (G.S.); cciofi@unime.it (C.C.)

**Keywords:** anechoic chamber, microwaves, minehunter vessels, shielding coating, shielding effectiveness

## Abstract

Testing the shielding effectiveness of materials is a key step for many applications, from the industrial to the biomedical field. This task is very relevant for high-sensitivity sensors, whose performance can be greatly affected by electromagnetic fields. However, the available testing procedures often require expensive, bulky, and heavy measurement chambers. In this paper, a cost-effective and reliable measurement procedure for testing the shielding effectiveness of materials is proposed. It exploits a lab-scale anechoic shielded chamber, which is lightweight, compact, and cost-effective if compared to the available commercial solutions. The measurement procedure employs a vector network analyzer to allow an accurate and fast characterization setup. The chamber realization phases and the measurement procedure are described. The shielding capability of the chamber is measured up to 26 GHz, whereas the performance of commercial shielding coatings is tested to demonstrate the measurement’s effectiveness.

## 1. Introduction

Until the 20th century, the only electromagnetic emissions on the Earth were due to natural phenomena such as the radiation from the sun, the atmosphere and the Earth itself. However, the continuous and impressive development of new electronic devices and telecommunication systems increased both the quantity and diversity of the electromagnetic emissions in terms of radiated power and frequency [1,2,3]. Although most of the electromagnetic emissions were intentional and beneficial for society, the coexistence of such a huge number of electromagnetic sources resulted in the onset of electromagnetic interferences (EMIs), which should be carefully considered in order to avoid electromagnetic compatibility (EMC) issues where EMIs interfere with the proper operation of other electronic devices [4,5]. The scientific community has always been prudent concerning the possible effects on human health; several examples have seen the possible effects of EMIs on test equipment, both at low and high frequencies, and for various applications from the biomedical to the industrial field [6,7,8,9,10,11,12,13].

EMI has a relevant impact on the performance of high-sensitivity sensors [14,15,16]. As an example, electrostatic shielding is a standard procedure for minimizing the capacitive coupling of soil sensors [15]. However, many EMI-sensing studies do not verify the effectiveness of the employed shielding. Moreover, sensors exploited in electronic warfare systems need to be protected from intentional electromagnetic attacks, thus requiring electromagnetic shielding [16].

It is worth noting that the problem, which is under the continuous supervision of the competent authorities, can be addressed and mitigated in a threefold way: (a) by suppressing the emission at the source, (b) by making the coupling path less efficient, (c) by making the receiver less susceptible to the emission [17].

Although the first line of defense is to suppress the emission as much as possible at the source, often this is not a feasible solution due to the intentional nature of the emission, or to the need to deal with EMI sources already present in the market and consequently in the environment.

Therefore, developing lightweight and cost-effective EMI-shielding materials is necessary to mitigate EMI pollution [18,19,20].

The basic concept behind the shielding effect involves the implementation of EMI-reflection and -absorption processes by means of a suitable material which in turn protects the shielded device from radiation [21,22]. This task can be fulfilled by exploiting conductive materials; to this aim, metal-based enclosure is probably the best-known type of EMI-shielding due to its excellent EMI-shielding effectiveness. However, metals present some limitations related to their weight, rigidity, vulnerability to corrosion and cost, which makes them unsuitable for many applications [23]. On the other hand, conductive polymer composites have shown their effectiveness, and are largely used as shielding materials due to their low cost, strong resistance to corrosion, and light weight [24,25]. Conductive polymer composites usually incorporate conductive fillers in a polymer matrix, thus approaching the electrical conductive properties of the hosting material [26,27].

In view of the availability of shielding materials, measurement procedures and tools are required to properly test their properties. To this aim, different kinds of test methodologies are reported in the scientific literature. In [28], the shielding effectiveness of planar materials has been measured by means of a method based on the ASTM D4935-18 standard [29]. It exploits an absorber box which mitigates the known issues of the ASTM D4935-18 standard. As an example, the 1.5 GHz upper frequency limit has been extended to 18 GHz. Even in the study of Tamburrano et al. [30], a similar technique has been implemented to extend the frequency up to 18 GHz. Moreover, in [31], an alternative method is presented for the SE measurement of planar materials with nonconducting surfaces. In particular, this method overcomes the edge termination problems by absorbing edge-diffracted energy. The results obtained are similar to those obtained using the ASTM D4935 method. Moreover, the method in [31] exploits a simple setup and is cost effective.

Different methods to measure radiated emission exploit reverberation chambers [32]. As an example, in [33], the shielding effectiveness of different materials has been measured in a nested reverberation chamber up to 6 GHz.

In this paper, a measurement setup aimed at testing the shielding effectiveness of material sheets is proposed. The idea has been fueled by the need for a specific application, i.e., the test of shielding panels for minehunter vessels. Indeed, minehunter vessels are required to be highly electromagnetically shielded to prevent the activation of the very sensitive detonator of the mine. The setup proposed in this study employs a lab-made anechoic and shielded chamber, working from 500 MHz to 26 GHz. Although shielded anechoic chambers are commercially available, they are usually very expensive. Through this paper, the realization steps are disclosed in order to make the chamber realization procedure reproducible. The problem of self-realizing anechoic chambers has received a good amount of interest within the scientific literature, with some papers exploiting anechoic chambers for similar purposes, but often with a lower maximum operating frequency [34,35,36,37]. Moreover, these works are usually devoted to the characterization of antenna parameters or electromagnetic compatibility tests, whereas the purpose of the proposed chamber differs completely from these applications. The measurement procedure exploits a vector network analyzer (VNA) to allow for an accurate and fast characterization. For the same purpose, microwave signal generators in combination with spectrum analyzers are frequently used in the literature. However, a VNA is able to sweep the incident wave across frequency, thus enabling faster measurement and minimizing the probability of errors. The shielding effectiveness measurement is based on a differential procedure. A face of the chamber is left open to hold the sheet of the material being tested. Therefore, two measurements of the S21 scattering parameter are performed, with the open face of the chamber covered once by the shielding material being tested and then with the untreated one. Finally, the shielding effectiveness can be obtained by the ratio of these two measurements or by their difference, taking into account that the magnitude of scattering parameters is usually provided in decibels (dB).

Both the shielding effectiveness of the chamber and the performance of commercial shielding coatings have been tested. This work has many cross-cutting applications in addition to the already mentioned ones. As an example, military vessels, vehicles and also satellites benefit from a shielding structure and are able to protect themselves from jamming, and in general from electronic warfare systems [38]. Many shielding accessories are available in the market to protect the human body from external electromagnetic sources, such as shielded clothes to preserve the human body and shielded windows and walls for residential use, or to protect confidential industrial or high-security data.

## 2. Construction of the Anechoic Shielded Chamber

**Figure 1:**
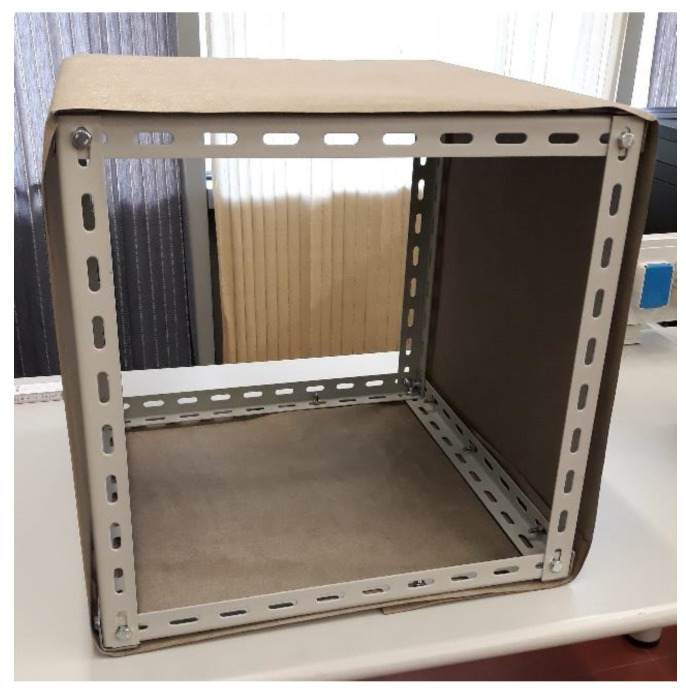
Picture of the anechoic chamber during the first realization step.

In this section, the construction of the anechoic chamber is discussed. The structure is composed of a 50 cm × 50 cm × 50 cm aluminum skeleton. These dimensions have been chosen because they fit the size of the shielding panels to be tested, i.e., 50 cm × 50 cm × 0.5 cm. The skeleton has been covered by a proper shielding material, the 3027-217 Flectron Nickel/Copper Polyester Nonwoven by Saint-Gobain S.p.A., which allows one to obtain a high shielding effectiveness with a very low weight. In Figure 1, a picture of the initial construction step of the chamber is shown.

**Figure 2:**
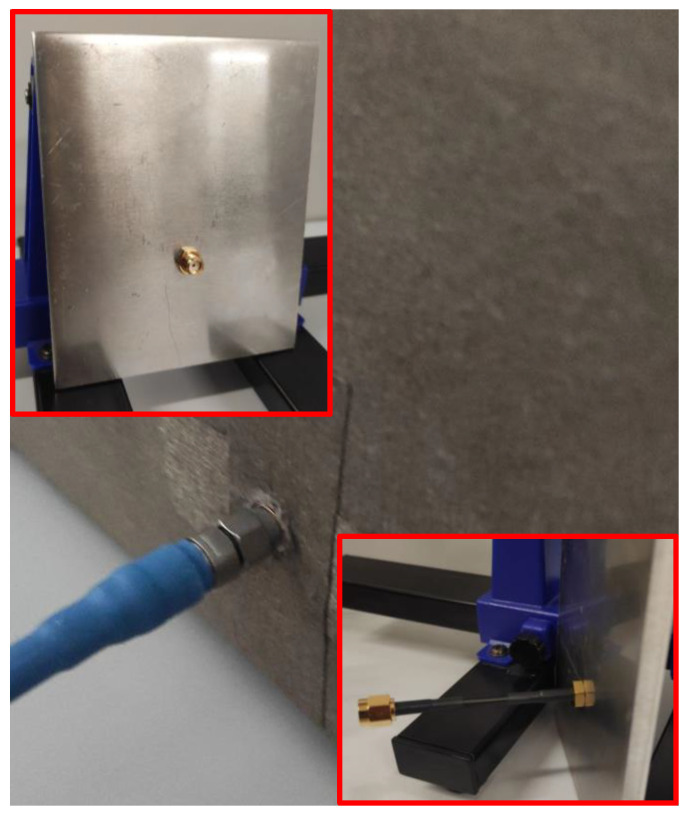
Picture of rear side of the chamber, with the detail of the panel connector in the insets.

Some very critical points in an anechoic chamber, deserving particular care, are the apertures. Although there should not be apertures in the chamber, the antenna inside the chamber must be connected to the instruments outside; thus, a small hole is required to make a path for at least one coaxial cable. In order to preserve the shielding properties, particular attention has been paid to this task. In particular, a steel sheet with a thickness of 5.5 mm was introduced in the rear face of the chamber. A 3.5 mm coaxial panel-connector, suitable to work up to 26 GHz, has been mounted on the steel sheet. By using shielding adhesive tape, it was finally covered with the shielding textile without leaving open slots, thus minimizing leakage. In Figure 2, the rear side of the chamber with the detail of the panel-connector is shown.

**Figure 3:**
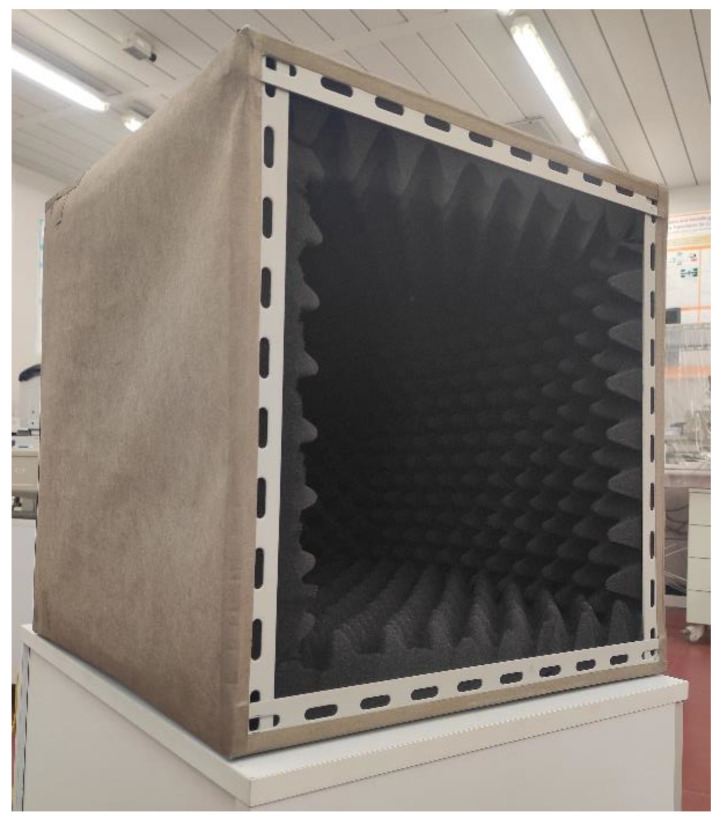
Picture of the shielding anechoic chamber.

The inside of the chamber has been covered by the anechoic material EA-PF3000-XX by Leader Tech. Inc. (Tampla, FL, USA). It is dielectrically loaded polyurethane, shaped in a pyramidal foam to have a smooth transitional impedance via the use of cones, thus providing the required reflection loss level. Figure 3 shows a picture of the realized anechoic chamber.

As mentioned in the Introduction, a face of the chamber is left open to hold the sheet of material being tested. However, in order to preliminarily measure the shielding effectiveness of the chamber itself, it needs to be closed; thus, a removable shield and anechoic cover has been designed and realized in the same way as the chamber. In order to ensure a stable closing of the chamber and to minimize leakage, neodymium super magnets have been used to tightly connect the chamber with the cover.

The total raw-material cost of the chamber is about €1500.00, thus about one order of magnitude lower than the cost of a commercial anechoic chamber of similar dimensions, where the design costs have a great impact. In the next section, the details concerning the measurement setup and the related performance of the chamber will be shown.

## 3. Measurement Setup and Performance of the Chamber

It is known that, considering an N-port network, where $V_{n}^{+}\;$ is the amplitude of the voltage wave incident on a port *n* and $V_{n}^{-}$ is the amplitude of the voltage wave reflected from a port *n*, the scattering matrix [S] can be defined in terms of the incident and reflected voltage waves, as shown in (1) [39]

(1) $$S_{ij}={\left.\frac{V_{i}^{-}}{V_{j}^{+}}\right|}_{V_{k}^{+}=0\;for\;k\neq j}$$

where *i* and *j* are the port numbers.

By considering a two-port network, it is possible to write the scattering parameters in terms of the incident and reflected power wave amplitudes *a* and *b*. In particular, the $S_{21}$ parameter is expressed in (2):

(2) $$S_{21}={\left.\frac{b_{2}}{a_{1}}\right|}_{a_{2}=0}$$

where the condition $a_{2}=0$ can be straightforwardly obtained by closing port 2 with a matched load. 

The shielding effectiveness, *SE*, of the chamber can be obtained by considering the ratio of the magnitude of the incident electric field, ${\vec{E}}_{I}$, to the transmitted electric field, ${\vec{E}}_{T}$, or the ratio of the magnitude of the incident magnetic field, ${\vec{H}}_{I}$, to the transmitted magnetic field, ${\vec{H}}_{T}$, or, alternatively, the ratio of the incident power, ${\vec{P}}_{I}$, to the magnitude of the transmitted power, ${\vec{P}}_{T}$ [20].


(3) $$SE=\frac{{\vec{E}}_{I}}{{\vec{E}}_{T}}=\frac{{\vec{H}}_{I}}{{\vec{H}}_{T}}=\frac{{\vec{P}}_{I}}{{\vec{P}}_{T}}$$

**Figure 4:**
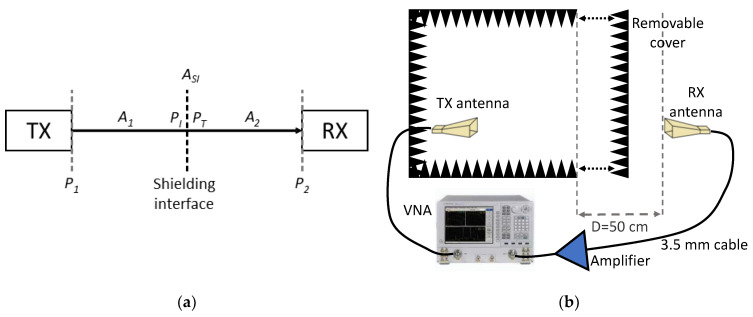
(**a**) Block diagram and (**b**) schematic representation of the measurement setup.

A block diagram of the setup is shown in Figure 4a. In particular, $P_{1}$ and $P_{2}$ are the powers generated by the transmitter and received from the receiver, respectively. They represent port 1 and port 2 of a VNA. $A_{1}$ and $A_{2}$ are the free-space attenuations before and after the shielding interface and $A_{SI}$ is the attenuation caused by the shielding interface, which can be written as in (4).


(4) $$A_{SI}=\frac{1}{SE}$$

By observing that the square module of $S_{21}$ is equal to the ratio of the received power to the transmitted power, it is possible to write $S_{21}$ alongside the shielding interface, i.e., the removable cover, $S_{21}^{shield}$, as in (5)

(5) $${\left|S_{21}^{shield}\right|}^{2}=\frac{P_{2}^{shield}}{P_{1}}=\frac{A_{2}P_{T}^{shield}}{P_{1}}=A_{2}A_{1}A_{SI}$$

where $P_{2}^{shield}=A_{2}P_{T}^{shield}$, and ${\;P}_{T}^{shield}=A_{1}A_{SI}P_{1}$.

$S_{21}$ can be presented without the shielding interface, $S_{21}^{open}$, as in (6)

(6) $${\left|S_{21}^{open}\right|}^{2}=\frac{P_{2}^{open}}{P_{1}}=\frac{A_{2}P_{I}}{P_{1}}=A_{2}A_{1}$$

where $P_{2}^{open}=A_{2}P_{I}$, and ${\;P}_{I}=A_{1}P_{1}$. This is because without the shielding interface, $A_{SI}$ can be considered equal to 1.

Therefore, *SE* can be measured by exploiting the scattering parameters, as in (7)

(7) $$SE={\left(\frac{\left|S_{21}^{open}\right|}{\left|S_{21}^{shield}\right|}\right)}^{2}={\left.S_{21}^{open}\right|}_{dB}-{\left.S_{21}^{shield}\right|}_{dB}$$

where $S_{21}^{open}$ and $S_{21}^{closed}$ are $S_{21}$ measured with the chamber open and closed, respectively.

The measurement setup is represented in Figure 4b. It is well known that a source-free environment would be very beneficial to perform shielding or EMC tests, avoiding the detrimental effect of external signals on the measurement’s effectiveness. However, the additional signals in the environment usually have very narrow bands compared to the measured bandwidth (500 MHz–26 GHz), e.g., tens of MHz within the 2.4 GHz Wi-Fi band, as much as can be considered a single scattered point which would be easily removed by averaging the measurement.

In particular, a VNA E8364A made by Agilent Technologies, Inc. (Santa Clara, CA, USA), working from 45 MHz to 50 GHz, has been used to straightforwardly measure $S_{21}$.

Indeed, although the powers of interest here can be also measured by using a microwave spectrum analyzer, it does not provide the option to sweep across the entire frequency range, thus requiring individual measurements, adjusting the frequency center for each tone sent from the signal generator.

Different transmitting (TX) and receiving (RX) antennas have been used to fulfill the whole bandwidth of interest, from 500 MHz to 26 GHz.

The far-field region boundary $R_{FF}\;$ can be calculated according to (8) [40].

(8) $$R_{FF}=2\frac{D^{2}}{\lambda }$$

where λ is the minimum wavelength of the lower frequency range.

The maximum dimension of the transmitting and receiving antennas are 10 cm and 29 cm, respectively. Therefore, in the worst case of the lower-frequency bound of 500 MHz, the far-field regions start at 29 cm and 3.33 cm, respectively. Due to the chamber dimension, and according to the setup of Figure 4, both antennas have always been placed at a distance higher than these limits.

Finally, two microwave amplifiers have been employed to increase the level of the signal; this is particularly important for the case of the measurement with the shielding panel. In particular, the amplifier 310 by Sonoma Instrument, working up to 2 GHz with a gain of around 32 dB and a gain flatness of 0.5 dB, and the 83,018 A by Agilent Technologies, Inc., working up to 26 GHz, with a gain higher than 27 dB at 20 GHz and 23 dB at 26 GHz, have been used.

**Figure 5:**
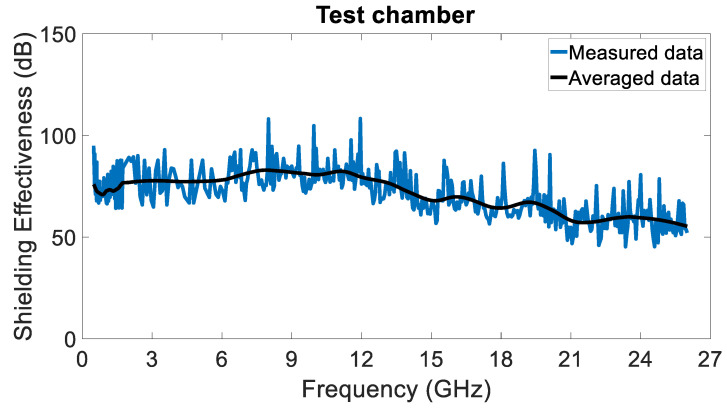
Shielding effectiveness of the anechoic chamber. Raw measured (blue line) and averaged (black line) data.

To measure the SE of the chamber, first, the reference measurement is performed in the absence of the shielding cover, i.e., with the chamber open; afterwards, the measurement is repeated with the shielding cover. The ratio (subtraction in dB) between the $S_{21\;}$ is extracted and shown in Figure 5, thus representing the total shielding effectiveness of the shielding and anechoic chamber, as illustrated in Figure 4. Due to the noisy nature of the measurement, both the measured raw and the averaged data are shown.

It is worth noting that, as a preliminary check, the sensitivity of the setup, i.e., the minimum measurable power, has been tested by performing different measurements while decreasing the VNA output power. As long as the effect of the output power decrease has been observed in the received power. The setup worked linearly, thus setting the dynamic range and validating the observed shielding effectiveness.

## 4. Test of Commercial Coating

The shielding effectiveness of panels, treated with commercial coating, has been measured and the results are reported in this section. 

As stated in the Introduction, the main application of this kind of panels consists in the shielding of minehunter vessels.

Two different composite panels have been considered and filled with basalt or glass fibers. The single-skin laminates have been prepared by infusion at INTERMARINE SpA (Sarzana, La Spezia, Italy). Both materials have been painted by using the commercial coating MAX54 by YSHIELD GmbH & Co. (Ruhstorf an der Rott, Germany), and CuPro-Cote by Less EMF Inc. (Latham, NY, USA). The first is an acrylic-based paint, enriched with micrometric graphite and carbon black (>40 wt%). The second is a latex-based paint, filled with micrometric copper particles (47 wt%). Both paints have been dissolved in a solvent (acetone) to morphologically characterize the present fillers, using a scanning electron microscope (SEM) (FEI, QUANTA FEG 450, ThermoFisher Scientific, Waltham, MA, USA) operating under high vacuum and 20 kV, equipped with Energy Dispersive X-ray Spectrometry EDS (EDAX, Ametek, Tokyo, Japan).

**Figure 6:**
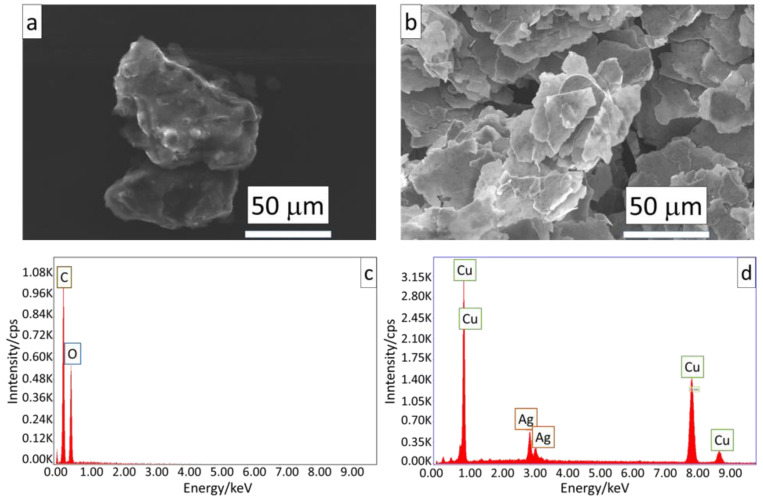
SEM images and EDS spectrum of MAX54 (**a**,**c**) and CuPro-Cote (**b**,**d**) fillers.

As shown in Figure 6, both types of fillers exhibit a flake morphology (Figure 6a,b), facilitating easy electrical continuity within the matrix. As confirmation of this, for MAX54 paint, only C and O (attributed to partial oxidation of the graphite) peaks are identified through EDS analysis (Figure 6c), while for the CuPro-Cote coating, the spectrum reveals the presence of Ag in addition to the Cu peaks (Figure 6d).

**Figure 7:**
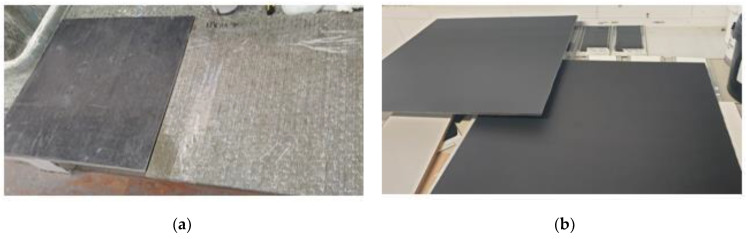
(**a**) Non-treated and (**b**) treated basalt and glass fiber panels on the left and right, respectively.

All investigated laminates have been coated with a layer of 2 mils, 50 μm of paint. Clearly, different behaviors are expected, not only depending on the type of coating but also on the treated material. Indeed, due to their better absorption capacity, the panels based on basalt fiber are expected to perform better than the ones based on glass fibers. Figure 7 shows the non-treated and treated basalt and glass fiber panels.

The measurement of the SE of the panel being tested can be obtained in a similar way, i.e., by measuring the $S_{21}$ parameter once with the non-shielding panel and then with the shielding panel, to finally calculate the ratio between the two measurements, or the difference in dB.

It is worth noting that the SE of the chamber itself must be higher than the SE of the panel being tested. Indeed, if the panels have a higher SE, the receiving antenna placed outside the chamber will receive the largest signal contribution from the chamber faces rather than from the panel being tested. As a matter of fact, this is not the case for the proposed setup; this might be easily verified based on the obtained results. Certainly, if the SE of the panel being tested shows values comparable to the SE of the chamber itself, the measured results might be considered dubious. In fact, if the SE of the panels being tested were higher, it could not be measured due to the higher leakages of the chamber itself.

**Figure 8:**
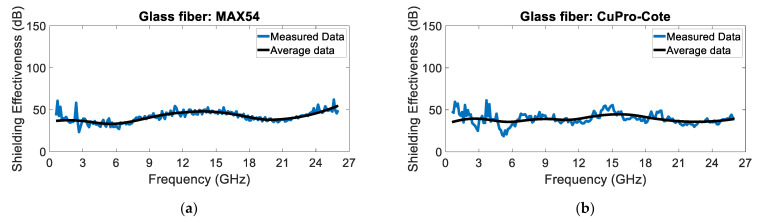
Glass fiber panels treated with (**a**) MAX54 and (**b**) CuPro Cote coatings.

**Figure 9:**
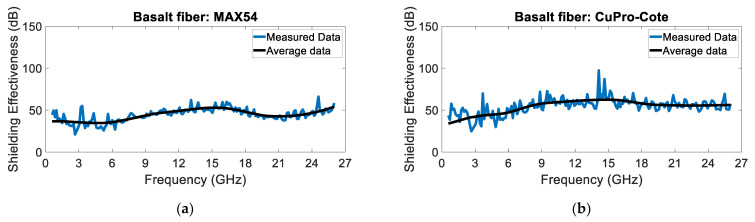
Basalt fiber panels treated with (**a**) MAX54 and (**b**) CuPro-Cote coatings.

Figure 8 and Figure 9 show the shielding effectiveness of the glass and basalt fiber panels, respectively. The measured SE is lower than the SE of the chamber; thus, the measurement can be considered valid. As expected, the basalt fiber panels show a higher SE, particularly at higher frequencies. Moreover, the CuPro-Cote coating’s performance is slightly better than the MAX43’s performance.

**Figure 10:**
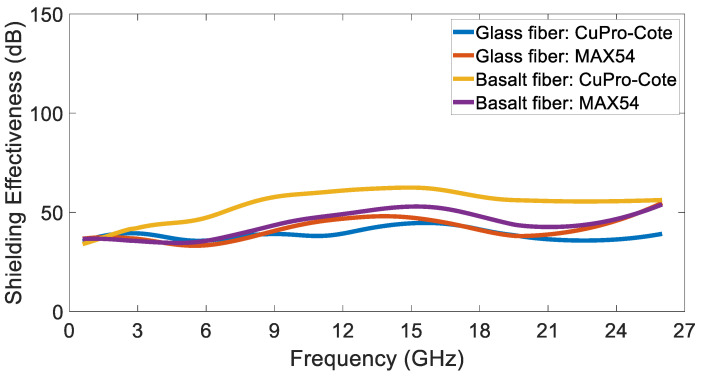
Shielding effectiveness of the tested coatings: glass fiber Cu-Pro-Cote (blue line), MAX54 (orange line), basalt fiber CuPro-Cote (yellow line) and MAX54 (purple line).

Figure 10 highlights the difference in shielding effectiveness between the tested materials. The performance of the glass fiber materials can be considered quite comparable, except for in the higher frequency range, i.e., from 21 GHz to 26 GHz, wherein the MAX54 fiber shows a higher attenuation. On the other hand, the basalt fiber CuPro-Cote exhibits a considerably higher shielding effectiveness throughout all of the frequency range.

## 5. Conclusions

This contribution dealt with a measurement procedure aimed at testing the shielding effectiveness of coated panels. The proposed setup exploited a lab-made anechoic shielded chamber whose realization steps have been disclosed. The cost-effectiveness and the ease of the measurement procedure are among the main advantages here. The shielding capability of the chamber was measured up to 26 GHz, whereas the performance of commercial shielding coatings was measured by testing different materials and paintings.

## Data Availability

Data are contained within the article.
